# Positive psychological characteristics in patients with metabolic syndrome associated with prospective changes in diet and anthropometric factors

**DOI:** 10.1371/journal.pone.0236693

**Published:** 2020-09-01

**Authors:** Cecilia Cesa Schiavon, Eduarda Marchetti, Fernanda Oliveira Ayala, Gabriela Loewe, Júlia Bauer, Fernanda Michielin Busnello, Caroline Tozzi Reppold

**Affiliations:** Psychological Assessment Laboratory, Department of Health Sciences, Federal University of Health Sciences of Porto Alegre (UFCSPA), Porto Alegre, Brazil; Università degli Studi di Milano, ITALY

## Abstract

The prevalence of metabolic syndrome (MetS) is increasing worldwide, and diet therapy plays a key role in treating this disease. Since most patients show difficulties in adhering to nutritional interventions, research on the association of positive psychological characteristics with greater engagement in physical health is relevant to this field. The present study aimed to evaluate the association between positive psychology attributes (optimism, hope, self-esteem, positive/negative affect and life satisfaction) and changes in diet quality and anthropometric parameters of individuals with MetS who received nutritional counseling. The study assessed 63 patients at a nutrition outpatient clinic. Anthropometric parameters and 24-hour food recall data (for evaluation of the Brazilian Healthy Eating Index—Revised–BHEI-R) were collected at the first visit and subsequent return visit (on average five months later). Psychological data were collected at the first visit using validated and standardized scales. The results were adjusted in relation to the depression scores of the patients, which were evaluated using the Beck Depression Inventory-II (BDI-II). Changes in anthropometric factors and in the BHEI-R were assessed, and their associations with the psychological attributes were investigated. The results indicated that positive affect and hope were associated with improvement in the BHEI-R scores (Cohen effect sizes -0.65 and -0.58; p = 0.012 and 0.025, respectively). A significant association was also observed between optimism and a reduction in abdominal circumference (Cohen effect size 0.56; p = 0.031). The associations remained significant even after adjusting for the BDI-II scores (p = 0.022, p = 0.037 and p = 0.05, respectively). No statistically significant associations were observed for the other attributes assessed.The study suggests that some attributes may have a greater influence on the nutritional treatment of MetS and that future studies should be conducted in order to enable effective multidisciplinary interventions to treat MetS.

## Introduction

Metabolic syndrome (MetS) is a set of interrelated factors that leads to cardiovascular disease and diabetes and is an important clinical and public health problem. These factors include dyslipidemia, high blood pressure, elevated triglyceride and low-density lipoprotein levels and obesity, mainly with central adiposity [1]. MetS is becoming increasingly prevalent worldwide and is estimated to affect approximately 20% to 25% of the global adult population, largely due to the increase in obesity and sedentary lifestyles over recent decades [2]. MetS doubles the probability of death and triples the probability of heart attack or stroke compared to that in people without the syndrome [1,3]. A systematic review of the prevalence of MetS in Brazil indicated a weighted average of 29.6% (range: 14.9%–65.3%) across ten selected cross-sectional studies [4].

According to the World Health Organization [5], a small set of risk factors accounts for the majority of deaths from chronic noncommunicable diseases, including smoking, excessive alcohol consumption, physical inactivity and poor diet. The main risk factor specifically for MetS is overweight/obesity, which seems to precede the onset of other risk factors for the syndrome [6].

Thus, the treatment and prevention of MetS and obesity follow similar patterns and are considered major clinical and public health challenges worldwide [7]. In this context, diet quality plays a key role in preventing MetS and its individual components [8]. Proper treatment can lead to a positive prognosis and good quality of life. However, studies have documented that patients have exhibited significant resistance to adhering to one or more of the self-management recommendations, such as medication use, physical activity and healthy eating [9,10].

Therefore, psychological factors can play a vital role in adherence to treatment. The literature presents ample evidence that depressive symptoms and anxiety are associated with non-adherence to diet, physical activity, medication and other secondary prevention measures [11,12]. However, the literature has also recently investigated positive mental health indicators as predictors of general health and longevity, including subjective well-being (SWB), optimism and self-esteem, among others [12].

The study of these constructs is called positive psychology (PP) and aims to broaden the focus of psychological studies, which have traditionally focused on risk factors for physical and mental illnesses [13]. Although also rooted in earlier humanistic psychology, PP began in 2000 and has grown significantly since then in several countries [14], especially in the context of health assessment. Seligman [15], a leading PP researcher, published an article titled “Positive Health” in which he specifically proposed that physical health studies take a positive perspective by investigating personal and social attributes that lead to positive outcomes in improving indicators of health, well-being, disease prevention and engagement in health-promoting situations.

However, it is emphasized that studies concerning the interference of positive psychological aspects in the context of metabolic syndrome are still scarce and have been relatively neglected in the literature. Some studies were found on diseases related to MetS but not to the syndrome itself. Recent reviews and meta-analyses have demonstrated that SWB is a protective factor against chronic diseases and mortality [16–18]. Likewise, Diener points out, based on several studies, that SWB is one of the variables with the greatest impact on physical health and that its mechanisms of action involve changes in health behaviors, the immune system and the cardiovascular system [19].

For example, Boylan and Ryff [20] evaluated a representative sample of middle-aged adults in the United States and found cross-sectional and longitudinal associations between well-being and MetS components (abdominal circumference, systolic and diastolic blood pressure, triglycerides, HDL cholesterol and fasting glycemia) and found that life satisfaction (LS) (p = 0.005) and positive affect (PA) (p = 0.009) predicted fewer MetS components as well as a lower risk of meeting the diagnostic criteria in fully adjusted models. The results did not change when adjusted for depressive symptoms, nor were they moderated by age, sex, race or socioeconomic status. The associations were stronger between SWB and abdominal circumference, HDL cholesterol and triglycerides. Therefore, according to the authors, body composition and diet may be the most relevant targets for SWB interventions.

In addition to SWB, other positive psychological attributes such as optimism, hope and self-esteem are considered important variables that protect against chronic disease, although they have been less studied in the context of physical health [21,22]. In the literature, these attributes are associated with greater engagement in appropriate health behaviors, such as adopting a healthy diet [23,24].

Considering the data presented, it is important to study positive psychological attributes in the context of MetS, especially regarding their impact on the nutritional treatment of the syndrome. Thus, the present study aimed to evaluate the association between positive personal indicators and the response to the nutritional treatment of patients with metabolic syndrome. The study specifically investigated the relationships among attributes studied by positive psychology (positive/negative affect, life satisfaction, optimism, hope and self-esteem) and improvements in the diet quality and anthropometric parameters of Brazilian individuals with MetS receiving nutritional counseling at a hospital outpatient clinic. It should be noted that diet quality was assessed using the Brazilian Healthy Eating Index—Revised (BHEI-R) as it measures several dietary risk factors for chronic diseases while simultaneously assessing and monitoring diet at an individual or population level [25]. In agreement with previous evidence, the hypothesis is that individuals with better positive psychology indicators show an improvement in diet quality and anthropometric factors upon their return visit to their nutritionist compared to their previous individualized nutritional counseling session.

## Materials and methods

### Sample inclusion criteria

This study included individuals who received treatment at the nutrition outpatient clinic of a public hospital in southern Brazil. According to the selection criteria, the subjects needed to be on their first outpatient visit, be cognitively able to understand the nutritional guidelines and answer the questionnaires of the study and have a confirmed diagnosis of MetS based on the biochemical, clinical and anthropometric data available in the patient files. The diagnostic criteria used were those adopted by the National Cholesterol Education Program’s Adult Treatment Panel III (ATP III). The literature presents several definitions of MetS, which vary somewhat in the clinical cut-off points that define risk, with ATP III being the most commonly used definition for studies on psychological factors and metabolic syndrome [20]. According to this definition, at least three of the following risk factors must be present to confirm the diagnosis: central obesity (Abdominal Circumference (AC) > 102 cm for men or > 88 cm for women); triglycerides ≥ 150 mg/dL; HDL cholesterol < 40 mg/dL in men or < 50 mg/dL in women; blood pressure ≥ 130 mm Hg systolic or ≥ 85 mm Hg diastolic; and fasting glucose ≥ 100 mg/dL.

### Instruments

#### Socioeconomic, clinical and anthropometric questionnaire

A questionnaire was used to assess information relevant to the sample, including age, sex, schooling, clinical comorbidities, monthly income and anthropometric data. The information was collected by the staff at the nutrition outpatient clinic. Abdominal circumference, weight and height were measured according to the standard procedures recommended by the World Health Organization [26]. Height and weight were measured using a Welmy^®^ digital scale with a stadiometer. Abdominal circumference was measured using an inelastic anthropometric tape at the midpoint between the ribs and the iliac crest. All anthropometric data were collected by the nutrition outpatient clinic team.

#### Brazilian Healthy Eating Index—Revised (BHEI-R)

Dietary consumption was measured using a 24-hour recall (24HR). Household measurements were converted into SI units of measure, and nutritional value was calculated with the Nutrition Data System for Research (NDSR, 2016 version). The resultswere used for classification by the BHEI-R, which was validated for the Brazilian population by Previdelli et al. [25]. The BHEI-R is a dietary index comprising 12 components, nine of which refer to food groups based on recommendations of the Food Guide for the Brazilian Population, in which higher consumption receives a higher score. The components for total fruit (including fruit and natural fruit juice); whole fruit (excluding fruit juice); total vegetables; dark green, orange and leguminous vegetables; total cereals (including cereals, roots and tubers); and whole grains have a maximum score of five points. The components for milk and dairy products (including soy derivatives), oils (monounsaturated and polyunsaturated fats, oils from oilseeds and fish fat), meat, eggs, and legumes have a maximum score of ten points. Two other components evaluate sodium and saturated fat specifically on a ten-point scale. Finally, the component that evaluates solid fats, alcoholic beverages and added sugar has a maximum score of 20 points. These last three components reflect an unhealthy diet. Thus, lower consumption receives a higher score. More information on the use of the BHEI-R can be found in the paper by Previdelli et al. [25].

#### Psychological scales

*Beck Depression Inventory II–BDI-II*. This inventory consists of a questionnaire with 21 multiple-choice questions and is intended to assess mood. Each item is scored on a scale of 0 to 3. The inventory was developed by Aaron Beck and has been standardized for Brazilian Portuguese; it has good internal consistency indicators and ample evidence of validity [27]. The instrument was applied at the beginning of the first interview with the patient to evaluate possible interference in the answers to the scales. The results of the other scales were adjusted to correct this factor since individuals with depression may have attention, memory and interpretation disorders related to emotionally relevant information [28]. The influence of the results of this scale on the change of the outcomes evaluated was also assessed.

*Positive and negative affect scale*. This scale comprises ten items that evaluate positive affect (PA) and ten that evaluate negative affect (NA). The items consist of adjectives with answer keys on a five-point Likert scale ranging from “not at all” to “extremely.” The scale was developed and validated by Zanon et al. [29], based on Watson and Clark’s [30] Positive and Negative Affect Schedule (PANAS) and has a two-dimensional structure; these dimensions add PA and NA items, respectively. The PANAS has been standardized for the Brazilian context and has adequate internal consistency indexes (with alphas of 0.83 and 0.77) as well as validity evidence based on internal structure and convergent validity.

*Life satisfaction scale*. This scale consists of five questions that are graded on a seven-point Likert scale, with responses ranging from “strongly disagree” to “strongly agree”. Studies have shown that the scale comprises a single factor and has high internal consistency (alpha = 0.87) and high test-retest reliability (r = 0.82) [29]. The instrument also features ample validity evidence based on internal structure and Brazilian standardization data.

*Brazilian version of the Revised Life Orientation Test (LOT-R)*. This test was developed to measure dispositional optimism and includes ten items: three statements about optimism (items 1, 4 and 10), three about pessimism (items 3, 7 and 9) and four distractors (items 2, 5, 6 and 8). Individuals indicate their level of agreement with the items on a five-point Likert scale with responses ranging from “strongly disagree” to “strongly agree.” Scores closer to 5 always indicate a higher degree of optimistic expectation. The LOT-R [30] is an abridged and revised version of the Life Orientation Test (LOT) [31]. Bastianello et al. [32] adapted, validated and standardized the instrument for Brazilian Portuguese, creating the Brazilian version of the LOT-R, which had an internal consistency of 0.80 and a single factor that accounted for 51% of the data variance.

*Adult Dispositional Hope Scale (ADHS)*. This scale assesses dispositional hope and contains 12 items distributed equally among agency factors, pathway factors and fillers. The responses are indicated on a five-point Likert scale and range from “totally false” to “totally true.” In Brazil, the original ADHS version by Snyder et al. [33] was adapted, validated and standardized by Pacico et al. [34], and an alpha coefficient of 0.79 was obtained. In the adapted instrument, the two-factor structure (pathways and agency) was not maintained, and the resulting factor structure was one-dimensional [35].

*Rosenberg’s self-esteem scale*. This scale has a one-dimensional measure consisting of ten statements that assess general self-esteem. The items are answered on a four-point Likert scale with responses ranging from “totally agree” to “totally disagree.” Hutz and Zanon [36] revised the adaptation, validation and standardization of the scale, which had been previously adapted based on preceding studies by Hutz. An analysis of its main components confirmed its one-factor solution, as was found in the previous Brazilian study and in the original American instrument. The internal consistency of the adapted scale (α = 0.90) was satisfactory.

#### Procedures

The present study has an observational longitudinal design. Subjects were assessed from 2015 until 2018 and its data were evaluated at two times in a distinct manner. At moment 1 (M1), first visit to the nutrition outpatient clinic, the interviewer read each question aloud so that the interviewees could better understand it. All of the instruments were applied at M1. Although the instruments were validated for self-application, it was found that the interviewer needed to mediate this adaptation in the pilot test of the present study, possibly due to the lower educational level of the sample compared to that used in the validation studies of the applied psychological scales.

At moment 2 (M2), which corresponded to the return of the patient to the outpatient clinic (i.e., the second visit to the outpatient clinic), only the 24HR for the BHEI-R, weight (kg), height (m) and abdominal circumference (cm) data were collected. Since the outpatient clinic visits always took place on Wednesdays, all patients were interviewed about the food they had consumed on the previous Tuesday, thus enabling the evaluation of a more usual weekly food consumption at both M1 and at M2. The subjects were evaluated for psychological constructs only at M1 since studies indicate that, although not immutable, these constructs have moderate temporal stability [33,37–41].

Nutritional recommendations are offered in both M1 and M2. The present study did not aim to evaluate the methodology of the nutritional recommendations strategies of the outpatient clinic where the data were collected. However, it should be clarified that the food behavior change methodology was based on the precepts of Bandura’s [42] Cognitive Social Theory, which adopts the perspective of acting for self-development, adaptation and change and that the nutritional guidance was provided in accordance with the First Brazilian Guideline for MetS Diagnosis and Treatment.

According to the anamnesis of each patient and based on the aforementioned guidelines, each diet plan was individualized and provided for sustainable weight reduction, varying from 5% to 10% of initial body weight. The first step in the design of the plan was to establish the needs of the individual based on nutritional assessment, including determination of body mass index and abdominal circumference. The eating plan provided a total caloric value compatible with obtaining desirable body weight. For obese subjects, the diet was hypocaloric, with a reduction of 500 to 1000 kcal in the total daily energy expenditure (DEE) predicted, aiming to promote weight losses from 0.5 to 1.0 kg/week. In addition, diet distribution, as recommended by the First Brazilian Guideline for Diagnosis and Treatment of Metabolic Syndrome [43], was 50 to 60% of the total caloric value (TCV) from carbohydrates and 20 to 30 g per day of dietary fiber. Total fats represent 20% to 30% of the TCV, with less than 10% saturated, up to 10% monounsaturated and up to 20% polyunsaturated fats.

At moment 2, which was an average of five months after moment 1, the results achieved and the difficulties experienced by the subjects were assessed. Nutritional counseling based on the same recommendations described above was provided again, and the diet was calculated using the same method.

### Statistical analyses

Due to the scarcity of data in the literature, a moderate effect size was used to calculate the sample size [44]. The calculation was performed using WINPEPI v.11.43 software. For a significance level of 5%, two-sided, 90% power and a minimum correlation coefficient of 0.4 for diet quality variation (delta) in relation to the anthropometric parameters and test scores for the psychological attributes, the resulting minimum sample size was 62.

Aiming to evaluate the change in the values ​​between the moments analyzed for the IQD-R score and the anthropometric factors, the delta, symbol Δ, was used. The delta calculation was performed by the difference between moment two (M1) and moment one (M1).

The analyses were performed using SPSS v.21.0 software. The Shapiro-Wilk test was used to evaluate the normality of the quantitative variables. Student’s t-test for paired samples was used to compare the means at M1 and M2, and the Wilcoxon Signed Rank Test was applied in cases of asymmetry. All the results of the psychological scales were dichotomized by the median scores. To compare the means of the anthropometric and BHEI-R variables according to the dichotomized psychological scales, Student’s t-test for independent samples was applied, and the effect size was checked with the Cohen formula. According to Cohen [45], values < 0.5 indicate a minor effect, while values between 0.5 and 0.79 indicate a moderate effect and values > 0.8 indicate a major effect. Analysis of covariance was used to control the confounding effect of the BDI-II results on the relationship of the psychological data with the change in BHEI-R score and anthropometric factors.

To control for confounding factors, a linear regression model with backward elimination was applied. The criterion for the entry of a variable in the multivariate model was that it had a p value <0.20 in the bivariate analysis.

### Ethics procedures

The project was duly approved by the ethics committee for research on human beings at the institutions that took part in the study. This study was carried out in accordance with the recommendations of the Human Research Ethics Committee of Irmandade Santa Casa de Misericórdia de Porto Alegre (ISCMPA) and the Human Research Ethics Committee of the Federal University of Healthcare Sciences of Porto Alegre (UFCSPA) and was approved under protocols 724,805 and 760,631, respectively. All subjects provided written informed consent in accordance with the Declaration of Helsinki.

## Results

The sample consisted of 63 subjects. The mean participant age was 55.3 ± 14.1 years, 68% (n = 43) of the subjects were female and 84% (n = 53) were white. Of the participants, 46% (n = 29) had less than eight years of schooling, and 59% (n = 36) reported incomes between three and five times the minimum wage. Regarding comorbidities, 73% (n = 46) had arterial hypertension, 76% (n = 48) had diabetes mellitus and 48% (n = 30) had dyslipidemia.

Scores on the BHEI-R, which is separated into 12 components, indicated a significant increase in total grain consumption from M1 to M2 (p = 0.038), an average of five months later, as described in Table 1.

**Table 1:** Comparison of Brazilian Healthy Eating Index—Revised (BHEI-R) scores between evaluations at M1 and M2 in patients with metabolic syndrome, Porto Alegre, Brazil (n = 63).

	M1	M2	P
Food Groups / Nutrients	Md (P25 –P75)	Md (P25 –P75)	
Total grains	5 (3.8–5)	5 (4.1–5)	0.981*
Whole grains	0 (0–3.2)	0 (0–5)	0.038*
Total fruits	4.7 (0–5)	4.8 (0–5)	0.689*
Whole fruits	5 (0–5)	5 (0–5)	0.334*
Milk and dairy products	4.6 (2.2–8.0)	5.2 (2.6–9.1)	0.257*
Meats	10 (10–10)	10 (9.9–10)	0.977*
Total vegetables	5 (5–5)	5 (5–5)	0.429*
Dark-green, orange and leguminous vegetables	5 (5–5)	5 (5–5)	0.646*
Oils	10 (4.9–10)	10 (2.8–10)	0.366*
Saturated fats	7.8 (3.9–9.7)	8.4 (4.0–9.5)	0.777*
Sodium	1.9 (0.4–3.8)	2.2 (0–4.0)	0.954*
SoFAAS	17.3 (13.4–19.8)	17.7 (14.9–20)	0.351*
Final–mean ± Standard Deviation (SD)	68.4 ± 10.4	69.0 ± 10.2	0.680**

M1: moment one; M2: moment two; SoFAAS: calories from solid fat, alcohol and added sugar; Md: median; P25: 25th percentile; P75: 75th percentile

*Wilcoxon test

**Student’s paired t-test.

Nevertheless, the final BHEI-R score did not change significantly. Consequently, no change occurred in the anthropometric parameters evaluated, with a mean Body Mass Index (BMI) of 33.4 kg/m^2^ ± 6.0 at M1 and 33.2 kg/m^2^ ± 6.2 at M2 (p = 0.485) and a mean abdominal circumference of 106.6 ± 12.5 at M1 and 105.8 ± 13.4 at M2 (p = 0.173) (Table 2).

**Table 2:** Anthropometric factors between M1 and M2 of patients with metabolic syndrome, Porto Alegre, Brazil (n = 63).

Anthropometric variables	M1	M2	p*
	Mean ± SD	Mean ± SD	
**Total Sample**			
Weight (kg)	86.8 ± 18.3	86.3 ± 18.6	0.472
Body Mass Index (kg/m^2^)	33.4 ± 6.0	33.2 ± 6.2	0.485
Abdominal Circumference (cm)	106.6 ± 12.5	105.8 ± 13.4	0.173

M1: moment one; M2: moment two; BMI: body mass index; AC: abdominal circumference; SD: standard deviation

*Paired t-test.

Table 3 shows the score for each of the main psychological variables analyzed.

**Table 3:** Scores of the psychological variables applied to patients with metabolic syndrome, Porto Alegre, Brazil (n = 63).

Psychological variables	Mean ± SD	Md (P25-P75)
BDI-II	10.0 ± 8.7	8 (4–14)
Positive affect	35.3 ± 8.2	36 (29–40)
Negative affect	20.9 ± 8.1	20 (15–27)
Life satisfaction	26.1 ± 6.6	27 (23–31)
Self-esteem	33.8 ± 4.1	34 (31–37)
Optimism	26.4 ± 3.6	27 (24–30)
Hope	34.6 ± 4.9	36 (31–38)

SD: standard deviation; Min: minimum; Max: maximum; Md: median; P25: 25th percentile; P75: 75th percentile.

Tables 4–10 show the classifications of the psychological characteristics associated with the BHEI-R, BMI, AC and weight of the 63 MetS evaluated patients.

**Table 4:** Classification of positive affect (dichotomized at the median) associated with the BHEI-R, BMI, AC and weight of patients with metabolic syndrome, Porto Alegre, Brazil (n = 63).

Variables		Positive affect		
	≤ 36 points (n = 32)	> 36 points (n = 31)	Cohen effect size (d)	P*
	Mean ± SD	Mean ± SD		
Δ BHEI-R	-2.94 ± 11.2	4.26 ± 10.9	-0.65	0.012
Δ BMI	-0.16 ± 2.39	-0.25 ± 2.26	0.04	0.885
Δ AC	-0.73 ± 4.84	-0.79 ± 3.81	0.01	0.956
Δ Weight	-0.62 ± 5.96	-0.46 ± 5.94	-0.03	0.916

Δ: variation between the two times evaluated; BHEI-R: Brazilian Healthy Eating Index—Revised; BMI: body mass index; AC: abdominal circumference; SD: standard deviation

*independent samples t-test. P-values are from independent t-test where the null hypothesis is delta **=** 0.

**Table 5:** Classification of negative affect (dichotomized at the median) associated with the BHEI-R, BMI, AC and weight of patients with metabolic syndrome, Porto Alegre, Brazil (n = 63).

Variables		Negative affect		
	≤ 20 points (n = 33)	> 20 points (n = 30)	Cohen effect size (d)	P*
	Mean ± SD	Mean ± SD		
Δ BHEI-R	0.20 ± 9.74	1.04 ± 13.3	-0.07	0.774
Δ BMI	-0.58 ± 1.27	0.21 ± 3.05	-0.34	0.191
Δ AC	-1.15 ± 4.54	-0.31 ± 4.08	-0.19	0.447
Δ Weight	-1.56 ± 3.51	0.58 ± 7.64	-0.36	0.168

Δ: variation between the two times evaluated; BHEI-R: Brazilian Healthy Eating Index—Revised; BMI: body mass index; AC: abdominal circumference; SD: standard deviation

*independent samples t-test. P-values are from independent t-test where the null hypothesis is delta **=** 0.

**Table 6:** Classification of life satisfaction (dichotomized at the median) associated with the BHEI-R, BMI, AC and weight of patients with metabolic syndrome, Porto Alegre, Brazil (n = 63).

Variables	Life Satisfaction			
	≤ 27 points (n = 33)	> 27 points (n = 30)	Cohen effect size (d)	P*
	Mean ± SD	Mean ± SD		
Δ BHEI-R	-1.31 ± 10.8	2.70 ± 12.0	-0.37	0.169
Δ BMI	0.02 ± 2.78	-0.45 ± 1.67	0.20	0.431
Δ AC	-0.82 ± 4.37	-0.69 ± 4.34	-0.03	0.910
Δ Weight	-0.17 ± 6.92	-0.95 ± 4.60	0.13	0.605

Δ: variation between the two times evaluated; BHEI-R: Brazilian Healthy Eating Index—Revised; BMI: body mass index; AC: abdominal circumference; SD: standard deviation

*independent samples t-test. P-values are from independent t-test where the null hypothesis is delta **=** 0.

**Table 7:** Classification of optimism (dichotomized at the median) associated with the BHEI-R, BMI, AC and weight of patients with metabolic syndrome, Porto Alegre, Brazil (n = 63).

Variables		Optimism		
	≤ 27 points (n = 34)	> 27 points (n = 29)	Cohen effect size (d)	P*
	Mean ± SD	Mean ± SD		
Δ BHEI-R	-1.70 ± 11.4	3.29 ± 11.3	-0.44	0.086
Δ BMI	0.05 ± 2.02	-0.50 ± 2.62	0.24	0.352
Δ AC	0.35 ± 3.58	-2.01 ± 4.79	0.56	0.031
Δ Weight	0.15 ± 5.50	-1.35 ± 6.33	0.25	0.319

Δ: variation between the two times evaluated; BHEI-R: Brazilian Healthy Eating Index—Revised; BMI: body mass index; AC: abdominal circumference; SD: standard deviation

*independent samples t-test. P-values are from independent t-test where the null hypothesis is delta **=** 0.

**Table 8:** Classification of hope (dichotomized at the median) associated with the BHEI-R, BMI, AC and weight of patients with metabolic syndrome, Porto Alegre, Brazil (n = 63).

Variables		Hope		
	<36 points (n = 29)	≥ 36 points (n = 34)	Cohen effect size (d)	P*
	Mean ± SD	Mean ± SD		
Δ BHEI-R	-2.88 ± 10.3	3.57 ± 11.8	-0.58	0.025
Δ BMI	-0.16 ± 2.34	-0.24 ± 2.32	0.03	0.894
Δ AC	-0.75 ± 5.63	0.76–2.93	0.00	0.996
Δ Weight	-0.49 ± 6.52	-0.58 ± 5.41	0.02	0.953

Δ: variation between the two times evaluated; BHEI-R: Brazilian Healthy Eating Index—Revised; BMI: body mass index; AC: abdominal circumference; SD: standard deviation

*independent samples t-test. P-values are from independent t-test where the null hypothesis is delta **=** 0.

**Table 9:** Classification of self-esteem (dichotomized at the median) associated with the BHEI-R, BMI, AC and weight of patients with metabolic syndrome, Porto Alegre, Brazil (n = 63).

Variables	Self-esteem			
	≤ 34 points (n = 34)	> 34 points (n = 29)	Cohen effect size (d)	P*
	Mean ± SD	Mean ± SD		
Δ BHEI-R	-1.25 ± 11.1	2.77 ± 11.7	-0.35	0.169
Δ BMI	-0.01 ± 2.75	-0.43 ± 1.68	0.18	0.475
Δ AC	-0.63 ± 4.16	-0.90 ± 4.57	0.06	0.809
Δ Weight	-0.29 ± 6.91	-0.83 ± 4.54	0.09	0.718

Δ: variation between the two times evaluated; BHEI-R: Brazilian Healthy Eating Index—Revised; BMI: body mass index; AC: abdominal circumference; SD: standard deviation

*independent samples t-test. P-values are from independent t-test where the null hypothesis is delta **=** 0.

**Table 10:** Classification of BDI-II (dichotomized at the median) associated with the BHEI-R, BMI, AC and weight of patients with metabolic syndrome, Porto Alegre, Brazil (n = 63).

Variables	BDI-II			
	≤ 8 points (n = 34)	> 8 points (n = 29)	Cohen effect size (d)	P*
	Mean ± SD	Mean ± SD		
Δ BHEI-R	0.85 ± 10.3	0.31 ± 12.9	0.05	0.854
Δ BMI	-0.43 ± 1.62	0.06 ± 2.93	-0.21	0.401
Δ AC	-1.29 ± 4.72	-0.11 ± 3.77	-0.27	0.287
Δ Weight	-1.02 ± 4.53	0.03 ± 7.23	-0.18	0.486

Δ: variation between the two times evaluated; BHEI-R: Brazilian Healthy Eating Index—Revised; BMI: body mass index; AC: abdominal circumference; SD: standard deviation

*independent samples t-test. P-values are from independent t-test where the null hypothesis is delta **=** 0.

PA and hope were significantly associated with a change in the BHEI-R, using the student’s t-test for independent samples, indicating that patients with higher scores on these scales at M1 had a greater increase in their BHEI-R scores at M2 compared to M1, although the effect size for these variables was moderate. When predicting BHEI-R, variables with univariate p-values less than 0.20 were considered candidate predictors in a multivariate linear regression model with a backward elimination where the selected level of stay was 0.05, and even when the results were adjusted for patient mood (BDI-II score), the differences remained significant (p = 0.022 and p = 0.037, respectively). A significant association between optimism and AC was also observed, as patients who had higher LOT-R scores experienced a greater reduction in AC, even after adjusting for the BDI-II scale (p = 0.05), and the effect size for this variable was also moderate.

Variables that presented a p-value less than 0.20 in the bivariate analysis with the variation in the BHEI-R were inserted in a multivariate linear regression model with a backward extraction criterion. The results of the multivariate linear regression analysis by Backward criterion to evaluate factors independently associated with BHEI-R variation are presented in Table 11.

**Table 11:** Multivariate linear regression analysis by backward criterion to evaluate factors independently associated with BHEI-R variation of patients with metabolic syndrome, Porto Alegre, Brazil (n = 63).

Variables*	Regression coefficient (b)	95% CI	Β	P
Positive affect >36 points	5.77	0.29–11.3	0.27	0.039
Age	0.27	0.07–0.48	0.36	0.011
Years of schooling	0.95	0.16–1.75	0.33	0.020
Δ AC	-0.94	-1.57–-0.32	-0.39	0.004

Δ: variation between the two times evaluated; BHEI-R: Brazilian Healthy Eating Index-Revised; AC: abdominal circumference; B: standardized regression coefficient (values with range between -1 and 1, where the larger the coefficient, the greater the strength of the association)

*variables that were excluded from the final model: BDI-II, optimism and hope scale, sex, physical activity, DM, SAH and variation in BMI.

After controlling for confounding variables, BDI-II, positive, optimism and hope scale, sex, physical activity, DM, SAH and variation in BMI, age, years of schooling and variation of abdominal circumference, the following variables remained significant in the prediction of BHEI-R scores: positive affect scale, age, years of schooling, and variation in AC. Patients who scored more than 36 points on the positive affect scale had 5.77 higher BHEI-R scores from the beginning of the study to the end of follow-up.

For each one year increase in age, there was an average increase of 0.27 points in the total BHEI-R score from the beginning of the study over the follow-up period. For each additional year of schooling, there was an average 0.95 point increase in the BHEI-R from baseline to the end of follow-up. BMI, AC and weight were not significant predictors in the multivariate model.

Finally, for each 1 cm reduction in waist circumference, there was an average increase of 0.94 points in the BHEI-R score over the follow-up. For the variables BMI, AC and weight, no factor remained statistically significant after the multivariate model was finalized.

## Discussion

The results indicated a significant increase in the consumption of whole grains, which is an important factor for glycemic control in subjects with type-2 diabetes mellitus (which corresponded to 76% of the sample) [46]. As expected, the mean BHEI-R score (i.e., the diet quality of the sample) was lower than that of national samples since the development of MetS is associated with poor diet quality, thus confirming the need for studies that contribute to its improvement. The sample evaluated had mean BHEI-R scores of 68.4 ± 10.4 and 69.0 ± 10.4 at M1 and M2, respectively. Loureiro et al. [47] evaluated 195 adults aged 20 to 50 in Brazil’s Center-West region and found an average BHEI-R score of 75.2 (95% CI = 74.2–76.1). Ceccatto et al. [48] evaluated women with breast cancer and found a mean BHEI-R score of 77.9 ± 9.07 before and 77.7 ± 10.41 after they had received treatment for the disease. Silva et al. [49] evaluated the BHEI-R scores of 38 patients with MetS in Brazil and found that their mean index score was even lower than that found in the present study (53.5, 31.2–78.1), thus indicating a relationship between MetS and poor diet quality.

No significant change occurred in the anthropometric parameters. According to Robertson et al. [50], despite multidisciplinary efforts, resistance to weight loss in overweight and obese patients remains high, suggesting that the approaches that have been taken are insufficient and that traditional psychology has been relatively ineffective in managing weight. Faith et al. [51] attribute the difficulty in drawing conclusions about the importance of psychological attributes in this context to the fact that most studies have explored “simple associations” between weight and psychological characteristics and that attempts to delineate causal pathways are underutilized, despite the potential for advancing the current knowledge in this area, as this study aimed to do by approaching the subject longitudinally.

Possible factors that contributed to the minimal change in diet quality and, therefore, in anthropometric factors of the sample evaluated include age, schooling and the method of dietary assessment. The literature shows that older individuals tend to be more resistant to change and that schooling is proportional to one’s understanding of dietary guidelines [52]. It should also be noted that the perception of being in an environment of nutritional guidance may lead individuals to fail to mention foods considered to be nutritionally poor or to overestimate their consumption of foods considered to be healthy; additionally, obese people tend to systematically underestimate their dietary intake [53]. In addition to these factors, a recent review suggests that MetS and its components exert synergistic and independent effects on brain structures, thus accelerating brain aging and cognitive decline, which could interfere with the understanding of nutritional guidelines [54].

Nevertheless, it was possible to confirm the initial hypothesis that individuals with higher PP constructs showed improvements in their diet quality or abdominal circumference in three of the constructs evaluated (PA, hope and optimism), even when adjusting for the BDI-II (which was found to have a moderate effect).

PA represents the emotional dimension of SWB, which is generally a three-part structure. One of these parts concerns the cognitive judgment of LS. The other two correspond to affective components: positive affect and negative affect [19]. Although some studies have evaluated these factors in isolation and others have investigated overall well-being, they have generally shown similar results when health outcomes are assessed regarding MetS [55].

However, Grant et al. [56], after evaluating the relationship between life satisfaction (LS) and seven health habits (smoking, exercise, alcohol consumption, sun protection, fruit consumption, fat intake and fiber intake) in an intercultural study with 17,246 young adults, concluded that there were differences in the influence of LS depending on culture. Their study showed that LS was positively associated with healthy habits, except for alcohol consumption and dietary fiber intake, in the entire sample. However, in the sample from the United States and Western Europe (n = 10,603), fruit consumption was not associated with LS. Thus, LS was not relevant to eating habits in this sample, which matches the results obtained in the present study. Considering that the Brazilian diet is approaching US standards (with an increase in overweight, obesity and the consumption of ultra-processed foods [57], the data from the present study sample corroborate the results presented in the study cited above.

Both Ford et al. [58] and the present study found a significant positive association between a healthy diet and PA when investigating associations between affect and the consumption of foods typical of the Mediterranean region as opposed to Western diets in 9,255 Americans who answered a food frequency questionnaire. White et al. [59] also evaluated the relationship between PA, NA and food intake among 281 adults in New Zealand for 21 days. Using a food diary, the subjects reported how many servings they had consumed from five food groups: fruits, vegetables, chocolates and cookie sandwiches, salty snacks, and cakes. The authors concluded that consuming more servings of fruits and vegetables was positively related to a higher PA score.

The present study found optimism to be another variable related to nutritional treatment success. The analyses indicated that patients with higher optimism scores had greater reductions in abdominal circumference (d = 0.56, p = 0.031). It should be noted that abdominal circumference is a marker used to assess cardiovascular disease (CVD) risk according to the American Heart Association [1]. Among the metabolic changes associated with abdominal obesity (defined by abdominal circumference) that contribute to an increase in the occurrence of MetS, glycemic disorder is strongly associated with CVD risk [60].

Among the positive psychological attributes, optimism is cited in the literature as being the most strongly associated with reduced CVD risk. A systematic review conducted by Schiavon et al. [21] evaluated optimism and hope in relation to chronic diseases and found empirical evidence in this context. One of the mediating factors of this association was healthy eating habits [23]. A review conducted by Boehm and Kubzansky [24] investigated the association between positive constructs and CVD and presented several studies that indicate that optimism and well-being are related to the consumption of healthier foods. Furthermore, those authors stated that the causal direction cannot be determined since nearly all of the studies reviewed were cross-sectional. Thus, they suggested reviewing longitudinal studies such as the present research.

In addition to PA and optimism, hope was also a construct that had significant relationships with one of the outcomes evaluated, that is, improvement in the BHEI-R, optimism and hope were positively related; thus, associations with the same outcomes were expected. However, they are distinct concepts. In a meta-analysis that examined both constructs in a simplified manner, Alarcon et al. [61] stated that an optimistic person believes that his or her future will somehow be successful and satisfying–whether due to luck, the actions of others or their own actions. The hopeful individual, on the other hand, specifically believes in his or her own ability to ensure a successful and satisfying future. In this way, it can be supposed that the differences between these concepts could reflect the significant association of both attributes with different outcomes if hope was associated with improvement in diet quality and optimism was associated with improvement in AC but there was no association of optimism with improvement in diet quality. This could also have been observed if optimism was more related to other changes in healthy behaviors, such as improvement in the practice of physical activity, which may have been reflected in the CA and not been due to the improvement in the quality of the diet itself.

Likewise, Snyder and Lopez [62] proposed that hope is goal-oriented thinking composed of pathways and agency. In a study by Pacico et al. [34], the authors explain that pathways refer to the ability to create appropriate routes to achieve a desired goal. Agency is referred to as the motivating component that drives the search for a goal using pathways. Agency is described as the belief in one’s own ability to use pathways and to achieve proposed goals [34]. Thus, in the article titled “Hope Theory: Rainbows in the Mind,” Snyder [63] noted that people with high scores on the hope scale were able to use information about physical illness as a pathway to prevention efforts since hope is defined as the perceived ability to find pathways to desired goals and to motivate oneself to use these pathways through one’s thoughts.

Therefore, it can be inferred that such motivation, which is inherent to the construct, contributed to improving the BHEI-R scores of the individuals in this study who had the highest scores on the hope scale. In 2013, Nothwehr et al. [64] also explored the concept of hope and its association with diet, but in a different way; the authors evaluated the use of behavioral strategies such as serving size control or meal planning. The authors evaluated 178 patients at a primary health care clinic who had a BMI > 25. The analyses demonstrated a significant association (p < 0.05) between the total hope score and all of the behavioral strategy measures, concluding that the hope measures should be explored in the context of weight loss intervention given its predictive association regarding the use of behavioral strategies [64].

More recently, Scioli et al. [65] evaluated the positive contributions of hope to maintaining and restoring health and concluded that greater hope was associated with a stronger commitment to adopting a healthier diet. In their study, diet was assessed by asking the participants if they were eating at least four servings of fruits and/or vegetables a day and, if so, for how long. If they were not, they were asked if they intended to make this change and when they planned to do so. The authors did not evaluate diet specifically but used a structured questionnaire based on the transtheoretical model (TM). The TM presumes that positive behavioral transformations progress through five stages of change: precontemplation, contemplation, preparation, action and maintenance. The study concluded that individuals who had higher hope scores were more likely to be in the contemplation stage than the precontemplation stage. This suggests that hope may be a factor that causes individuals to direct their thoughts toward a healthier diet.

The results of the present study did not show a significant relationship between self-esteem and the outcomes evaluated. Nevertheless, the literature indicates that high self-esteem scores mediate coping strategies for physical illnesses, while low self-esteem can lead to inappropriate eating behaviors in an attempt to self-regulate negative emotions [66]. In fact, a study by Bonsaksen et al. [67] found that low self-esteem was associated with minor weight loss in obese people after they received nutritional counseling. It should also be noted that the sample evaluated in the present study had a similar mean score on the Rosenberg Self-Esteem Scale (33.80 ± 4.10) to that of a sample of similar ages that was evaluated in another study in southern Brazil (35.10 ± 4.10) [68]; therefore, this may not be a possible explanation for the lack of improvement in diet quality and, thus, in anthropometric parameters. However, there is still a gap in the literature regarding the predictive effect of self-esteem in the context of treating chronic diseases.

Finally, as previously mentioned, a complementary analysis was performed with the BDI-II results, which were associated with the outcomes evaluated, although the main objective of the BDI-II evaluation had been to correct possible confounding factors. Diet quality studies among psychiatrically diseased samples are largely lacking [69,70], but it is known that individuals with depression may have attention, memory and interpretation disorders related to emotionally relevant information [28], which could also contribute to decreasing their understanding of nutritional counseling and, consequently, adherence to treatment. However, no differences were observed among subjects with different BDI-II scores compared to the changes in the outcomes evaluated between the two evaluation moments in this study.

Somerset et al. [71] evaluated depression scores and adherence in a dietary weight loss intervention trial and concluded that there was a significant correlation (R = -0.38, p < 0.05) between BDI-II scores at baseline and the duration of participation in the trial. Subjects with a baseline BDI-II of 10 (moderate to severe depression symptoms) were more likely to drop out of the trial before 10 weeks (p < 0.001). In our study, we also had dropouts and patients with large intervals between consultations; there was a total of 39 dropouts.

Somerset et al. [71] discussed that the trial resignation phenomenon represents a significant burden on the development and delivery of effective weight loss intervention programs for a range of reasons. First, it imposes substantial upfront costs for research studies since baseline data are collected and subjects are then lost to follow-up. In addition to the added cost, this compromises the statistical analysis of trial outcomes. This situation is replicated in the clinical setting, where major investments in baseline assessment and intervention delivery is spent on patients who dropout. In essence, the evidence base for effective weight loss intervention is restricted to those patients who persist with treatment. Therefore, it is expected that more studies on positive psychological attributes can contribute in this sense, improving the follow-up in treatments referring to diet and many other factors.

Finally, the study has some shortcomings, primarily concerning the small sample size, which prevented a broader variety of statistical tests and analyses by sex, among other measures. However, as it sought subjects submitted to the same nutritional treatment methodology and considering the longitudinal nature of the study, a larger sample would render the study unfeasible due to time. More dietary information, based on a higher number of 24HRs, would also have provided more precise information on the dietary habits of the sample. Nevertheless, the authors understand that collecting such information in a way other than in person might have compromised the standardization of the data collected. Another shortcoming refers to the psychological scales employed, for which the validations were, in general, performed with populations with more schooling than that of the sample in this study. To minimize this possible bias, the interviewer read each question aloud in an attempt to obtain better comprehension of the sentences by the respondents. The reliability of the self-report method must be highlighted. As previously mentioned in the diet evaluation, this is also an issue with psychological data. Several areas of knowledge, such as psychology and nutrition, use self-reports in research, but it is known that there are limitations in this practice. Self-report does not guarantee the likelihood that behavior will be real and assured, according to Kohlsdrof and Costa Jr. [70], because (a) reported behavior differs from actual behavior when facts are not highlighted naturally; (b) the reported behavior is not real, but socially desired behavior, considering influences from the history of reinforcement of the individual; and (c) the participant does not understand the items that make up the instrument used in view of the distinctions between the participant and researcher.

## Conclusions

Studies that have assessed this relationship have done so by evaluating specific dietary components, such as fruit and vegetable consumption. However, diet quality goes beyond the consumption of specific food groups, as the present study has shown. Some constructs seem to be more relevant to engaging individuals in improving their diets and, hence, their anthropometric measurements.

As this is an observational study, its findings do not indicate causality. Therefore, further studies should be conducted to better understand the importance of these attributes with regard to adherence to nutritional guidelines. Considering that it is important to prevent the comorbidities associated with Mets that are difficult to treat, the constructive approach of positive psychology is currently proving to be a promising path to reach such an understanding.

Finally, it is noteworthy that the results of the present study may be implemented in future interventions, since the attributes are stable as long as they do not undergo intervention and there are currently promising documented results of interventions using positive psychology in a variety of environments, including hospitals [72].
